# Adolescent pregnant women’s health practices and their impact on maternal, fetal and neonatal outcomes: a mixed method study protocol

**DOI:** 10.1186/s12978-019-0719-4

**Published:** 2019-04-25

**Authors:** Tahere Hadian, Sanaz Mousavi, Shahla Meedya, Sakineh Mohammad-Alizadeh-Charandabi, Eesa Mohammadi, Mojgan Mirghafourvand

**Affiliations:** 1 Students’ Research Committee, Tabriz University of Medical sciences, Tabriz, Iran; 2 Women Reproductive Health Research Center, Tabriz University of Medical Sciences, Tabriz, Iran; 3 Member of South Asia Infant Feeding Research Network (SAIFRN), School of Nursing, Faculty of Science, Medicine and Health, University of Wollongong, Wollongong, Australia; 4 Social determinants of Health Research Center, Tabriz University of Medical sciences, Tabriz, Iran; 5 Department of Nursing, School of Medicine, Tarbiat Modares University, Tehran, Iran

**Keywords:** Health practices, Adolescent pregnant women, Mixed method

## Abstract

**Background:**

Considering that individuals’ health practices can affect the health of both mothers and babies, this study is designed to: (a) assess adolescent pregnant women’s health practices and their relationship with maternal, fetal, and neonatal outcomes; (b) explore the perception of adolescent pregnant women about their own health practices; and (c) recommend some strategies to improve adolescent pregnant women’s health practices during pregnancy.

**Methods/design:**

This mixed-method study with the sequential explanatory design has two phases. The first phase (quantitative phase) is a prospective study to assess the adolescent pregnant women’s health practices and its relationship with maternal, fetal, and neonatal outcomes who live in Tehran, the capital city of Iran. A cluster sampling method will be used to select 316 adolescent pregnant women who visit health centers in Tehran. The second phase is a qualitative study designed to explore the adolescent pregnant women’s perception of important aspects and factors of health practices that can affect their health outcomes. In this phase, purposive sampling and in-depth individual interviews will be conducted for data collection. The conventional content analysis approach will be employed for data analysis. In addition to literature review and nominal group technique, the findings of the qualitative and quantitative phases, will be used to recommend some strategies to support adolescent pregnant women to improve their health practices during pregnancy.

**Discussion:**

This is the first study looking into health practices in adolescent pregnant women which will be performed via a mixed-method approach, aiming to develop health practices improvement strategies. It is worth noting that there is no strategic guideline in Iran’s health system for improvement of health practices of adolescents. Therefore, it is hoped that the strategy proposed in the current study can enhance health practices of adolescents during pregnancy and ultimately improve their pregnancy and childbirth outcomes.

**Ethical code:**

IR.TBZMED.REC.1397.670.

## Plain English summary

Adolescent pregnancy is a public health concern that affects mothers, their children, and the broader community. Pregnancy and childbirth complications remain the leading cause of mortality and morbidity among female adolescents worldwide and can be influenced by lifestyle choices. The rate of adolescent pregnancy is increasing globally and due to recent changes in family planning policies in Iran, it is estimated that adolescent pregnancy will increase in the near future. The current study provides precise information about the health practices in Iranian adolescent pregnant women, and the factors related to them. This study is a mixed-method with the sequential explanatory design has two phases. The first phase (quantitative phase) is a prospective study to assess the adolescent pregnant women’s central and dispersion indices of health practices and its relationship with maternal, fetal, and neonatal outcomes who live in Tehran, the capital city of Iran. The second phase is a qualitative study designed to explore the adolescent pregnant women’s perception of important aspects and factors of health practices that can affect their health outcomes. The findings of the qualitative and quantitative study in addition to literature review and nominal group technique will be used to recommend some strategies to support adolescent pregnant women to improve their health practice during pregnancy. The strategy proposed by this study may be helpful in promoting health practices in adolescent pregnant women and improving pregnancy and childbirth outcomes in them.

## Background

Adolescents account for approximately 1.2 billion people worldwide, which is one-sixth of the world population [1]. The World Health Organization (WHO) defines an adolescent as any person between ages 10 and 19 [2]. Findings of the 2016 population census in Iran showed that out of 79,926,270 Iranian population, 11,147,381 were adolescents including 5,450,270 females [3]. Each year, approximately 16 million girls aged 15 to 19 years and 2 million girls under 15 years give birth, with 95% in low- and middle-income countries [4]. Each year, 10% of babies are born to adolescent mothers, who account for 23% of maternal mortality and complications [5]. Despite the lower rate of adolescent pregnancy in Iran compared to the global rate (nearly 7%), it is expected that changes in human population planning policies will result in an increase in this rate in following years [6].

Adolescent pregnancy is a public health concern that affects both the adolescent mother, her child, and the broader community [7]. Pregnancy and childbirth complications are the leading causes of mortality among adolescents aged 15–19 years worldwide [2]; however, the majority of these complications are preventable [5, 8]. The adverse maternal and neonatal outcomes among adolescent pregnant women and their babies include abortion, preterm birth, anemia, postpartum depression, pregnancy hypertension, preeclampsia and eclampsia, puerperal endometritis, systemic infection, maternal mortality, low birth weight, stillbirth, and neonatal mortality [9–16].

The health practices of pregnant women can affect maternal and fetal health, and pregnancy outcomes [17, 18] and they include: avoiding tobacco, alcohol, and other illegal substances [19, 20], avoiding high-risk sexual behaviours [21], having a healthy diet for appropriate maternal weight gain during pregnancy [22], regular exercise, adequate rest and sleep [23, 24], oral hygiene [25], regular prenatal cares, and acquiring knowledge about pregnancy and childbirth [19, 26].

According to the literature review, no study into health practices of Iranian adolescent pregnant women, either quantitative or qualitative, and no relevant mixed-method study was found internationally. Only one cross-sectional study has been conducted in Iran on pregnant women at gestational ages between 33 and 41 weeks. The findings of this study showed that maternal-fetal attachment and health practices during pregnancy have a significant positive relationship with neonatal outcomes. Results from few studies into pregnancy and childbirth outcomes in adolescent pregnant women in Iran showed higher prevalence of adverse outcomes of pregnancy and childbirth among this age group. A systematic review study in Iran (2017) showed that adolescents are at a higher risk of pregnancy and childbirth complications, which may disrupt Iran’s national development objectives (lowering maternal mortality and morbidity). As a result, development of a health practice improvement strategy specific to adolescents may have a significant role in achieving national development goals. Given the high risk of adolescent pregnancy, maternal and neonatal complications, and positive effect of health practices on health status and reduction in adverse maternal and neonatal outcomes, pregnancy health practices should be promoted, specifically in adolescents. To this end, identification of the status of such practices is essential. It is worth noting that the Iranian Health System lacks a strategic guideline on the improvement of health practices of adolescents.

## Study aim

This study aimed to determine the factors related to the health practices of adolescent pregnant women and their relationship with maternal, fetal, and neonatal outcomes. Moreover, health practices and their relevant factors will be explained from the perception of adolescent pregnant women. Then, an improvement approach to health practice in adolescent pregnant women will be developed.

The specific objectives are: 1) Determination of the health practices score of adolescent pregnant women visiting health centers in Tehran-Iran; 2) Determination of the relationship between health practices with some maternal outcomes (preeclampsia, type of delivery, anemia, pregnancy depression, and maternal weight gain) in adolescent pregnant women visiting health centers in Tehran-Iran; 3) Determination of the relationship between health practices and some neonatal outcomes (neonatal anthropometric indicators, low birth weight, preterm birth, and SGA) in adolescent pregnant women visiting health centers in Tehran-Iran; 4) Determination of the relationship between health practices and some fetal outcomes (abnormalities and stillbirth) in adolescent pregnant women visiting health centers in Tehran-Iran; 5) Determination of the relationship between socio-demographic characteristics and health practices in adolescent pregnant women visiting health centers in Tehran-Iran; 6) Determination of the relationship between health practices and maternal-fetal attachment in adolescent pregnant women visiting health centers in Tehran-Iran; 7) Exploration of the perception of adolescent pregnant women with high and low performance of health practices; 8) Exploration of the perception of adolescent pregnant women and the relationship between health practices and maternal, fetal, and neonatal outcomes; and 9) Provision of improvement strategies to health practice in adolescent pregnant women visiting health centers in Tehran-Iran.

## Methods/design

### Study design

**Fig. 1:**
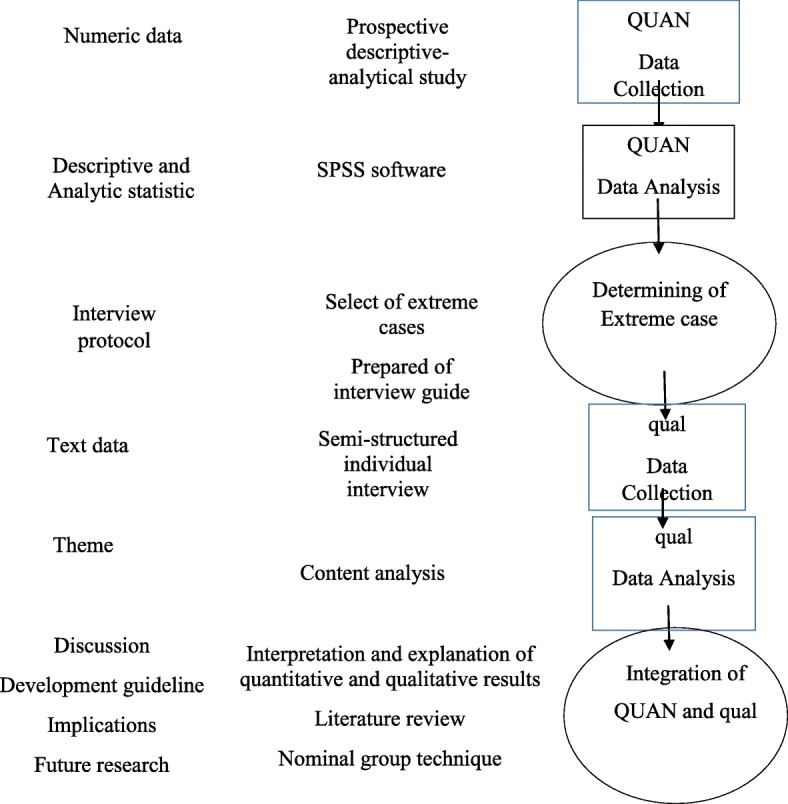
Study diagram

This study uses a mixed method with an explanatory sequential approach for data collection and analysis. The mixed-method paradigm is based on the principles and logic of pragmatism. According to this paradigm, a mixed use of qualitative and quantitative approaches results in a better understanding of the problem [27, 28]. The quantitative data will be collected in the first phase of the study. The second phase will include the collection and analysis of qualitative data. Then, qualitative and quantitative findings will be mixed in the stage of data interpretation and development of improvement strategies to health practices in adolescent pregnant women (Fig. 1).

### Phase one: quantitative study

First, a prospective descriptive analytical study will be conducted to evaluate factors related to health practices in adolescent pregnant women and the relationship between health practices and maternal, fetal, and neonatal outcomes among a population of Iranian adolescent pregnant women. The target population are adolescent pregnant women visiting health centers in Tehran-Iran.

### Sample size and sampling method

The sample size was calculated to be 158 based on the Madahi et al study [29], health practice variable, SD = 11.14, precision (d) of 0.02, α = 0.05, m = 123.57, and power of 90%. Regarding the use of cluster sampling, the final sample size was determined to be 316 by considering the design effect of 2.

This study will be conducted in health centers in south Tehran affiliated with the Tehran University of Medical Sciences, health centers in west and northwest Tehran affiliated with Iran University of Medical Sciences, and health centers in east and northeast Tehran affiliated with Shahid Beheshti University of Medical Sciences. In the cluster sampling, one-fourth of health centers will be randomly selected, using https://www.random.org. Then, a list of adolescent pregnant women at the gestational age between 18 and 22 weeks will be prepared using their medical profiles in each center. Then, the required samples from each health center will be determined using a proportional method and randomly selected. The researcher will explain the project to them via telephone (obtained from their medical records) and the eligible women will be invited to participate in the study.

### Inclusion criteria

The eligible participants are 18 to 22 week pregnant Iranian women who are between 10 and 19 years old; without any medical conditions such as diabetes, hypertension, kidney, thyroid, and heart diseases; and live in Tehran.

### Exclusion criteria

Women with multiple pregnancies, obstetric problems (such as placenta previa), history of bleeding in the current pregnancy, and plausible movement in the next four months will be excluded from the study. Moreover, exposure to stressful events during or one month before the pregnancy will lead to exclusion.

### Scales and data collection

Quantitative data will be collected using the inclusion-exclusion checklist, socio-demographic and obstetrics characteristics questionnaire, Health Practice Questionnaire (HPQ), Edinburgh Postnatal Depression Scale, Maternal-Fetal Attachment Scale, and maternal, fetal, and neonatal outcome checklist. In addition, data will be collected through face-to-face interview or from medical records of the participants.

The socio-demographic and obstetrics characteristics questionnaire will include age, spouse age, educational attainment, socioeconomic status and etc.

The HPQ, designed by Lindgren in 2003, includes 34 items scored on a five-point scale anchored by “1 = never,” “2 = almost never,” “3 = sometimes,” “4 = almost always,” and “5 = always.” It measures the following six factors in pregnant women: balance between rest and activity, preventing disease and injury, diet, avoidance of harmful medicine and opiates, follow up health status and acquiring knowledge about pregnancy and childbirth. The maximum and minimum scores are 170 and 34, respectively. Higher scores reflect better practice. Items 5, 6, 7, 8, 12, 21, 22, 23, 24, 25 are inversely scored. The Farsi version of this instrument was applied on a group of pregnant women in Sirjan-Iran in 2014 and its reliability was measured using the intraclass correlation and internal consistency; where the intraclass correlation was 0.81 and Cronbach’s alpha was 0.83 [29].

The Edinburgh Postnatal Depression Scale was designed by Cox et al. (1987) to measure depression during and after pregnancy. This instrument includes 10 items with four response options, ordered from lowest-to-highest severity (Items 1, 2, and 4) and highest-to-lowest severity (Items 3, 5, 6, 7, 8, 9, 10). Items are scored between 0 and 3 based on the severity of the symptoms. The total score ranges from 0 to 30. Mothers with scores higher than the threshold of 12 have varying degrees of depression. In Iran, validity and reliability of this instrument were confirmed by Montazeri et al. and they obtained the Cronbach’s alpha of 86% [30].

The Cronley’s Maternal-Fetal Attachment Scale is a self-report instrument which evaluates the mother’s sense of attachment to her fetus. This 23-item scale is scored based on a five-point Likert scale anchored by “5 = Absolutely Yes,” “4 = Yes,” “3 = Not Sure,” “2 = No,” and “1 = Absolutely No.” Only Item 22 is scored inversely as “1 = Absolutely Yes,” “2 = Yes,” “3 = Not Sure,” “4 = No,” and “5 = Absolutely No.” The minimum and maximum scores are 23 and 115 respectively, and a higher score indicates greater attachment. Cronley reported the reliability of α = 0.85 for this instrument based on the internal consistency [31]. In Iran, validity and reliability of this instrument were confirmed by Abbasi et al. and they obtained the Cronbach’s alpha of 80% [32].

The maternal, fetal, and neonatal outcome checklist includes items on preeclampsia, type of delivery, anemia, pregnancy depression, maternal weight gain, maternal-fetal attachment, fetal abnormalities, stillbirth, neonatal anthropometric indices, low birth weight, preterm birth, and SGA.

The validity of the socio-demographic and obstetrics characteristics questionnaire and maternal, fetal, and neonatal outcome checklist will be determined using content and face validity. Reliability of the health practices, depression, and maternal-fetal attachment questionnaires will be determined through test-retest in 20 adolescent pregnant women and obtaining internal consistency (Cronbach’s alpha) and ICC (Intraclass Correlation Coefficient (. To determine the reliability of hemoglobin and hematocrit tests, the first 10 samples will be delivered to the laboratory with two different names. Then, the correlation of results will be calculated.

### Data analysis

The quantitative data will be analyzed with SPSS-24. Sociodemographic and obstetrics characteristics and health practices will be described by frequency (percent), as well as mean (standard deviation) if the data are normally distributed or median (25 to 75 percentile) if they are not normally distributed. The relationship of health practices with maternal, fetal, and neonatal outcomes will be determined using the independent t and Pearson correlation tests in the bivariate analysis, and logistic linear regression adjusting the confounding variables in the multivariate analysis. The bivariate tests, including Pearson correlation, independent *t*-test, and one-way ANOVA, will be used to determine the relationship between socio-demographic and obstetrics characteristic with health practices. Then, the multivariate linear regression with backward strategy will be used to control confounding variables. The confounding variables will initially be controlled via inclusion and exclusion criteria. In the next stage, the multivariate tests (multivariate logistic regression and multivariate linear regression) will be applied.

### Phase two: qualitative study

Phase two is an exploratory qualitative study with a conventional content analysis approach to explore health practices in adolescent pregnant women in more detail.

### Sampling method

The extreme cases will be selected based on the overall mean score of the health practices obtained in the quantitative phase. Of that, women who obtain 10% of the lower and upper thresholds of the total health practices score will be selected as the extreme cases. The research participants will be selected through purposive sampling among extreme cases who have the tendency and ability to express their experience of health practices. Moreover, participants who differ from other participants in some variables, as well as those with unexpected findings will be interviewed.

### Data collection

Qualitative data will be collected using in-depth and semi-structured interviews, containing open questions. Before conducting the qualitative phase, the desired items in the interview guideline will be designed based on the findings from the first phase and the relevant factors. The mechanisms of obtaining valid data and focusing on research items will be reviewed by the research team. The interview will begin with a key question, “what health practices do you adopt for yourself and your child?” Then, the interview will continue by presenting other questions, such as “what factors facilitate health practices?” or “what factors inhibit health practices?” based on the participants’ responses. The interview will continue with more in-depth items, such as “what do you mean? Why? Can you explain further? Can you give an example?” to explore the depth of their experience. During the interview, the researcher will record nonverbal data of the participants, such as tone, facial expression, and position, in a specific sheet, along with the time and place of the interview. The sampling will continue until data are saturated.

### Data analysis

The qualitative data will be analyzed using qualitative content analysis with an inductive approach. In this approach, the data will be analyzed through frequent text reading to obtain a full understanding of it. Then, the texts will be read word by word to extract the codes. First, the objective words that contain the key concepts will be specified. The researcher continued digging the text by taking notes from the initial analysis until the major codes will be extracted. In this process, the code labels reflecting more than one key thought will be directly extracted and specified. Then, the codes will be categorized based on their difference and/or relationships. Ideally, 10–15 categories will be considered sufficient for categorization of a huge amount of data. This study uses an inductive content analysis based on the stages proposed by Graneheim and Lundman [33]. This method allows for extracting not only the explicit content of the texts, but also their implicit content with varying degrees of abstraction. Based on this method, the following five stages will be taken:

1. Transcribing the whole interview immediately after each session.
2. Multiple reading of the whole text to obtain a general knowledge of its content.
3. Dividing the text into semantic units, extracting a summary and coding them.
4. Categorizing the initial codes into classes and subclasses based on their differences and similarities.
5. Extracting the themes as the implicit concept and content of data.

### Integration of quantitative and qualitative data

To develop improvement strategies for health practices in adolescent pregnant women, a comprehensive literature review will be carried out with a supportive approach to improve such practices. Following this, the improvement strategies to health practices in adolescent pregnant women, along with the results from qualitative and quantitative studies will be delivered to 10–12 experts. Then, their feedback and comments will be taken into account, using the nominal group technique.

## Discussion

Adolescent pregnancy and childbirth are associated with adverse obstetrics, maternal, and neonatal outcomes [9–12]. Regarding the positive effects of health practices on health enhancement and reduction of maternal and neonatal complications [17, 29], they should be promoted during pregnancy, specifically among adolescents. To this end, the status of this practice should be identified. The current study provides precise information about the health practices in Iranian adolescent pregnant women, and the factors related to them. Data collection through qualitative and quantitative methods contribute to better understanding of health practices in adolescent pregnant women, and its relationship with maternal, fetal, and neonatal outcomes. The mixed-method approach focuses on Epistemological Pluralism. As a result, it supports the combination of opinions, approaches, and different, even contradictory, methods if they are helpful for understanding concepts [34].

The strategy proposed by this study may be helpful in promoting health practices in adolescent pregnant women and improving pregnancy and childbirth outcomes in them. Regarding the growing population of adolescents in the world, it is predicted that the global number of adolescent pregnancies will increase by 2030 [35]. It is also expected that recent changes in human population planning policies in Iran, aiming at promoting population growth policies and encouraging women to have 3 children before the age of 30, will increase this rate in future [6]. The development of health practice improvement strategies for promotion health practices in adolescent pregnant women will result in the improvement of pregnancy and childbirth outcomes.

## Abbreviations

EPDS: Edinburgh Postpartum Depression Scale
HPQ: Health Practice Questionnaire
MFAS: Maternal Fetal Attachment Scale
WHO: World Health Organization
